# Social classification and the changing boundaries of learning. A neopragmatic perspective on social sorting in digital education

**DOI:** 10.1080/17439884.2023.2219900

**Published:** 2023-06-05

**Authors:** Kenneth Horvath, Mario Steinberg

**Affiliations:** Educational Research, Zurich University of Teacher Education, Zurich, Switzerland

**Keywords:** Learning, sorting, classification, algorithms, French pragmatic sociology

## Abstract

Allowing learners to move across learning contexts in novel ways, digital tools play an increasingly central role for the formation of learning trajectories and identities. They thus presumably also affect dynamics of social sorting in education. Against this background, this article introduces a conceptual framework for unravelling dynamics of social sorting in digital learning environments. Inspired by French pragmatic sociology, we propose *classification* as analytical anchor point for disentangling the intricate interplays between educational technologies, learning situations, and wider moral and social orders. We present a ‘speculative inquiry’ into current AIED to demonstrate the added value this analytical perspective. We identify a hiatus between ‘inspired’ and ‘industrial’ logics of classification in current digital learning tools and environments that are likely to yield unwanted social sorting effects. A classification lens helps foreground social dynamics underlying such patterns, thus furthering our understanding of persistent patterns of disadvantaging in (digital) education.

## Introduction

Digital learning tools allow learners to cross the ‘boundaries of learning’ and move within and between different learning contexts in ways that would have been inconceivable just a few years ago. They hence play an increasingly important role for the formation of individual learning trajectories and identities. By implication, they thus also contribute to changing dynamics of social sorting in school education (Domina, Penner, and Penner 2017): learners from different social and cultural background presumably differ in how they engage with digital learning environments, in how well their learning biographies are compatible with digital learning tools, and how they are empowered to establish links between various learning contexts and their everyday lifeworlds.

Whether or not digital learning tools will affect learning pathways and subjectivities in an *equitable* manner is, however, an open question. On the one hand, there are hopes of empowering disadvantaged students by accounting for the individual needs and strengths of every individual learner (Buckingham Shum and Luckin 2019; Silseth 2012; Roth and Erstad 2016; Greenhow and Lewin 2016; Leander, Phillips, and Taylor 2010). On the other hand, there is an increasing awareness that digital learning tools might aggravate rather than alleviate existing disadvantages and thus reinforce existing social and pedagogical boundaries by algorithmically discriminating against specific groups of learners (Macgilchrist 2019; Williamson and Eynon 2020; Dixon-Román, Nichols, and Nyame-Mensah 2020; Ben Shahar 2017).

Obviously, *boundaries of learning*, *digital learning tools*, and *social sorting* are deeply interwoven, even if in unclear ways. It is this link that this article is concerned with. Our key concern is how to analytically grasp the intricate interplays between situations of learning, digital technologies, and social inequalities. Our objective hence is a conceptual one, following repeated calls for widening our analytical repertoire for researching the social foundations and implications of digital learning (Selwyn et al. 2020; Williamson and Eynon 2020; Macgilchrist 2021). Following Bechmann and Bowker (2019), we propose *classification theory* as possible anchor point for unravelling the sociocultural entanglements of digital learning. Our take on classification is inspired by French pragmatic sociology and, more specifically, Alain Desrosières’ ‘sociology of numbers’ (Desrosières 2011a, 2011b). The key characteristic of this analytical perspective is that it starts from underlying moral and epistemic orders, focusing on how widely shared ideas of what makes ‘good and fair school education’ translate into classificatory logics that inform the definition and shaping of everyday learning situations as well as the design of educational technologies. With view to digital tools, these classificatory logics inform, for example, what challenges and aspects of everyday learning processes software developers focus on, or what data and indicators they use for capturing and evaluating learning processes. Regarding situations of learning, classificatory logics leave their marks, among others, on the kind of learning environment that is envisioned for digital learning and on how involved actors assess the adequacy and fairness of learning processes. Classification thus forms a kind of interface and shared infrastructure to which actors from both the technological and the pedagogical field relate. Together, these technological and pedagogical design processes define the contexts in which individual learning trajectories and identities can be formed in more or less equitable ways.

As is discussed below, the productivity of a classification-lens is particularly obvious in the context of AI-based educational technologies (AIED). We therefore focus on this particular kind of learning tools in this article. The general idea is, however, more widely applicable since any educational technology inevitably relates to established educational logics of classification.

In the following, we first discuss the linkages between technologies, learning, and social sorting as they are envisioned in current debates on the boundaries of learning in the digital era. We then motivate our focus on classification and introduce three analytic heuristics that together define a classification-centred analytical framework: *conventional logics* of classification, *test designs*, and *situations of learning*. The analytical potential of this framework is illustrated by a ‘speculative inquiry’ (Macgilchrist, Allert, and Bruch 2020; Gulson, Sellar, and Taylor Webb 2022) into the social foundations and implications of AIED. Drawing on findings from existing scholarship and our own research, we identify a decisive hiatus between two opposing logics of classification dominating current AIED and the EdTech landscape more generally. We argue that this tension is likely to result in unwanted sorting effects. These sorting dynamics are particularly troubling because they are systematically invisibilized, both technologically and pedagogically. Building on this ‘speculative inquiry’, the concluding section discusses how the presented classification lens can further our understanding of the relations between the boundaries of learning and social sorting in digital education beyond AI-related EdTech.

## Social sorting and the boundaries of learning in the digital era

This article proposes classification theory as a conceptual framework for furthering our understanding of the social implications of digital learning and the concomitant changes in the boundaries of learning. Why should such a move be needed and useful? In a nutshell, we argue that classification defines a powerful anchor point for unravelling persistent dynamics of social sorting that emerge in the interlocking of learning environments, individual biographies and lifeworlds, and technological devices.

One prominent narrative regarding such sorting dynamics maintains that digital learning technologies can empower disadvantaged students in novel ways (Roth and Erstad 2016; Sefton-Green and Erstad 2017) by expanding, shifting, or even abolishing traditional boundaries of learning (Leander, Phillips, and Taylor 2010). There are many good reasons for this hope. Not only do the very nature of knowledge and the purposes of learning change in times of big data and deeply networked information societies (Säljö 2010); there is also a widely shared understanding that digital technologies can help organize new forms of life-long, life-wide, and life-deep learning (Bricker and Bell 2014; Bosch, Laubscher, and Kyei-Blankson 2021). Further, digital tools shall allow crossing informal and formal learning as well as online and offline learning in novel ways, creating learning environments that allow for hitherto unimaginable flexibility of learning trajectories (Kumpulainen, Mikkola, and Jaatinen 2014; Greenhow and Lewin 2016).

Less enthusiastic accounts are of course possible and plausible. This is particularly obvious in the case of AIED, which this paper concentrates on. AI-based algorithms are today already in use in formal and informal learning contexts. Applications of AI include intelligent tutoring systems in online and offline learning environments (Perrotta and Selwyn 2020), writing instruction and assessment assistants (Dixon-Román, Nichols, and Nyame-Mensah 2020), face recognition tools (Gulson, Sellar, and Taylor Webb 2022), detection of emotional states (McStay 2020; McStay and Rosner 2021), or classroom management software that provides teachers with immediate information on individual students’ learning progress and learning behaviour through interactive dashboards (Jarke and Macgilchrist 2021).

In the case of AIED, the promises of educational justice are imagined as being directly inscribed into the technologies themselves. AI-based learning tools shall enhance educational equity and fairness exactly by algorithmically adapting learning processes to the needs and interests of each and every individual student – they are believed to ‘increase equality of opportunity by fostering independent, flexible, reflective, teamworking individuals who have developed grit, tenacity and a sense of self-empowerment’ (see e.g. the critical account by Macgilchrist, Allert, and Bruch 2020, 76).

Such solutionist hopes of technologically solving complex social problems have of course been challenged many times (Morozov 2014; Nachtwey and Seidl 2020). Thus, critical education scholars have argued that AI-based educational technologies risk to reproduce or even exacerbate rather than alleviate existing educational disadvantages – thus linking learning pathways in an unforeseen and unwanted manner to learners’ out-of-school backgrounds and lifeworlds (see, e.g., Macgilchrist 2019; Williamson and Eynon 2020; Beer 2018; Dixon-Román 2016; Knox 2017; Knox, Williamson, and Bayne 2020).

These critical studies offer rich accounts of digital learning tools as powerful assemblages that are deeply entangled with political and social orders (Perrotta 2021; Williamson 2015, 2017a, 2017b). However, several conceptual and methodological challenges remain. One of the main conundrums concerns the question of *how to conceptualize the interplay of learning pathways that take shape across various learning contexts, digital technologies, and social sorting*: Through what mechanism and processes do social inequalities become effective in technologies and learning environments? How can we conceive of the entanglements of social orders of inequality, learning environments, and the mediating role of digital tools? In other words, how exactly can we explain that learning technologies ‘inherit sociopolitical relations’ and how is it that they become ‘sociopolitical forces shaping the potential transactions of teaching and learning’ (Dixon-Román, Nichols, and Nyame-Mensah 2020, 237)?

This article is motivated by the search for concepts and frameworks that allow to address this kind of questions. There are numerous points at which we may start disentangling these interplays. Drawing on new materialism, Dixon-Román, Nichols, and Nyame-Mensah (2020) conceptualize *data* as the analytical starting point to understand how social relations ‘inhere’ in digital learning platforms. Whitman (2020), in contrast, puts the analytical focus on *models* and *algorithms*: the way in which variables are treated either as *attributes* (assumed to be fixed and beyond the control of students, such as social class and gender) or *behaviours* (which students can morally be expected to change) can have important consequences for how social inequalities become effective and visible in digital learning contexts, thus relating the digital to the non-digital in specific and consequential ways.

In this paper, we follow yet another approach. Drawing on Bechmann and Bowker (2019), we propose to focus on *classification* as analytical anchor point (Bowker and Star 2000; Diaz-Bone 2016, 2017). We hence ask: How could classification theory help understand and unravel the interplay of digital learning and social sorting?

## The case for classification

The term classification refers to the ways in which social actors draw boundaries and make distinctions in a manner that can be expected to be accepted as relevant and fair by others. Classification theories typically address three interrelated aspects: (1) the concrete categories that are considered necessary to grasp social realities, (2) the moral and epistemic orders underlying these categories, and (3) the concrete processes of applying these categories in real-world situations (Bowker and Star 2000; Bourdieu 2002; Douglas 2011; Rosch 1983; Durkheim and Mauss 1903).

Why exactly should we choose classification – thus understood – as anchor point for reflecting and analysing how digital tools might reconfigure boundaries of learning and dynamics of social sorting? The basic intuition is particularly obvious in the case of AIED. After all, it is through algorithmically solving classification problems that algorithms red-flag critical learning trajectories, recommend learning content, assign tasks to individual students, diagnose learners’ emotional or motivational state, or relate information on learners across different learning contexts. Based on some (often implicit) theory of learning, students are sorted into latent or explicit categories or matched with other students, tasks, etc. (Al-Amoudi and Latsis 2019; Sahlgren 2021). In the case of AIED, the techno-pedagogical vision of *expanding* and *abolishing* the ‘boundaries of learning’ hence actually relies on sophisticated techniques of *drawing* and *enacting* boundaries. These boundary dynamics become socially consequential as soon as they are interlocked with processes of social sorting in school education (Domina, Penner, and Penner 2017; Horvath and Leemann 2021).

On a more theoretical level, the work by Bechmann and Bowker (2019) and others (cf. Bowker and Star 2000) suggests three main reasons for focusing on classification to understand the social implications of these boundary dynamics: First, classifications form a widely shared knowledge infrastructure (Bowker and Star 2000; Diaz-Bone 2016); for example, social actors routinely rely on established classifications when defining and distinguishing learning environments, evaluating what makes meaningful learning content, or thinking and talking about social inequalities in education. Second, this knowledge infrastructure is shared across different fields of practice. Classifications are not only *infrastructure*, but also define *interfaces*. For example, software development and pedagogical situations become interlaced through categories such as ‘at-risk-student’ or ‘low/high achievement’, with actors from both the technological and pedagogical field relying on some common notion of what defines success or failure in learning. Third, classification matters because it constitutes both social *visibility* and social *discrimination*. Our capacity to evaluate the social justice of learning environments and processes depends on having categories at hand for comparing groups of learners – and for making them visible as advantaged or disadvantaged. At the same time, these very categories might constitute biases in technologies and learning environments, for example when gender is used as a predictor for likely learning outcomes, thereby systematically biasing against girls irrespective of their individual potentials.

We propose an analytical framework for addressing these issues that is inspired by French pragmatic sociology (Boltanski and Thévenot 1999; Holmqvist 2020) and, more specifically, Alain Desrosières’ ‘sociology of numbers’ (Desrosières 2000, 2011b, 2011a, 2015, 2016). In his empirical and conceptual work, Desrosières combines a political economy of statistical classification with in-depth investigations of how social actors ‘on the ground’ make sense of and engage with statistical categories and information. In a nutshell, Desrosières conceives of *classification* as a special case of *quantification*. The design and implementation of classification entails the construction of *statistical chains* along which various actors become actively involved or passively affected by processes of turning social phenomena into numbers. *Coordination* along these statistical chains depends on *plural conventions* that allow to assess the relevance, quality, and fairness of the procedure.

The following section introduces this perspective in three steps which follow the theoretical architecture of Desrosières ‘sociology of numbers’ (and French pragmatic sociology in general). Three guiding heuristics follow from this perspective which we present in the following. In a first step, we introduce historically formed sociocultural *conventions* (heuristic 1) that actors rely on when engaging with classifications; in contexts of learning and teaching, this translates into widely shared ‘conventional’ understandings of what makes good and fair education (‘epistemic and moral orders’). Second, we discuss *tests* (heuristic 2) as analytical key to understand how such logics are applied; in educational contexts, this means to focus on what kind of digital tools and learning environments are designed and how they are implemented as ‘sorting devices’ in concrete contexts. Third, we emphasize the need to grasp (3) *situations* in which these tests develop concrete social consequences; in other words: we look at concrete learning situations in which individual learners shape their identities and trajectories in an interplay with the always already bounded and bordered learning contexts surrounding them. Our main argument is that *in their combination*, ‘conventions’, ‘tests’, and ‘situations’ provide a productive analytical framework for researching and reflecting dynamics of empowering and disadvantaging in current digital learning and education. We illustrate this potential with a speculative inquiry into AIED. This speculative inquiry builds on existing scholarship and findings from an ongoing research project on algorithmic sorting in education. In this project, we explore how actors in the technological and the pedagogical field envision and enact the role of algorithmic classification (most importantly those linked to Artificial Intelligence) in school education. To this end, we conduct case studies and focus group discussions.^1^

Our speculative inquiry leads us to (1) suspect a fundamental tension on the level of conventional classificatory logics in current digital education, (2) which is not adequately reflected on the level of technological devices and learning environments, (3) with troubling implications for how social boundaries become effective in concrete learning situations.

## Unravelling sorting dynamics in digital learning – a neopragmatic classification lens

### Conventions – or: identifying a hiatus of classificatory logics in digital learning

The notion of conventions plays a central role in Desrosières’ sociology of numbers. In line with French pragmatic sociology, Desrosières understands conventions as sets of rules that actors refer to in order to interpret, evaluate, and handle uncertain situations in a manner that is widely accepted as reasonable and acceptable (Lewis 1975; Batifoulier 2001; Diaz-Bone 2016). One of the main tenets of French pragmatic sociology is that in any concrete situation social actors have a plurality of such conventions at hand (Boltanski and Thévenot 1999, 2006). For Desrosières, these pre-existing conventions play a decisive role in processes of statistical classification. Without conventions, actors could not think and talk about the purposes of classification, the relevance of categories, or the adequacy of classification procedures (Desrosières, 2009). Conventions inform the choice of ‘equivalence principles’ – the target criterion on which a classification is built (Desrosières 2011b; Desrosières and Thévenot 1979). Conventions also influence how actors conceive of the political and practical role and relevance of categories and numbers (Desrosières 2015). Conventions, thus understood, define the moral and epistemic footing on which classifications are built, i.e., the basis for evaluating the quality and justice of classification (Diaz-Bone 2016).

How could this tenet of plural conventions help understand the interplay of digital learning and social sorting? In learning and education, as elsewhere, there is a plurality of conventional understandings available for assessing questions of quality and fairness. Derouet (1992) identifies four such currently dominant conventional orders which he denotes as ‘school worlds’ (Leemann and Imdorf 2019; Horvath, Steinberg, and Frei 2023): a civic, an industrial, an inspired, and a market-oriented school world. A civic school world is centred on the problem of promoting equality by educating a nation’s future citizens, an industrial school world is organized around the efficient and effective teaching of knowledge and skills needed for economic productivity, an inspired school world focuses on the idea of education and learning as creative process of self-fulfilment, and a market-oriented school world concentrates on success and profitability of investment into education.

Each of these school worlds defines different understandings of the nature and purposes of learning and education and comes with characteristic pedagogical perspectives, didactic approaches, organizational forms, implications for how to group and sort students, and preferences regarding fair and valid testing and evaluation of students’ learning trajectories. Each of these school worlds thus implies its own classificatory logic which informs judgments on what distinctions seem relevant, adequate, and fair in learning contexts, on how to define valuable and relevant learning content, on how to delimit and relate spaces of learning, and on how to evaluate the quality and justice of learning trajectories that take shape in these spaces (all of these being different forms of ‘classificatory work’).

It is against the background of these classificatory logics, anchored in ‘school worlds’, that software developers design algorithms, teachers create digital learning environments, or learners engage with digital learning tools. ‘Conventional logics of classification’ thus mark a mechanism through which a shared knowledge infrastructure becomes inscribed in digital learning settings. It is essential to note that these school worlds are only one aspect of the complex decision-making work that goes into educational technologies. Most importantly, they interact with the marketized context of platform economics that define the first and most pressing problem that EdTech entrepreneurs need to handle: acquiring funds and turning them into profit for themselves and investors (cf. Decuypere, Grimaldi, and Landri 2021; Komljenovic 2021; Williamson 2019). However, as soon as EdTech shall enter the educational field and be taken seriously as valuable learning tools, established understandings of educational quality and justice must be always *also* considered.

Turning to our ‘speculative inquiry’ into AIED technologies, the heuristic of conventions shifts attention to a conspicuous hiatus between two countervailing orders of classification. Aspects of both these logics have been noted by many other authors (e.g., Chang 2019; Sahlgren 2021; Rahm 2021): (a) a behaviourist logic that emphasizes the need and value of standardized testing of curricular knowledge and competencies and (b) an authenticity-centred logic that focuses on creativity, team working, and self-fulfilment.

A classification lens adds to findings from existing scholarship regarding these two logics by furthering our understanding of where this tension comes from, why it proves so persistent, and what consequences it might have. Seen through a classification lens, this often-noticed tension unfolds between ‘inspired’ and ‘industrial' school worlds and their respective logics of classification. In our own research, we observed these two opposing logics when studying narratives of AI in education in different online outlets (TED talks, blog posts, marketing material) (Horvath, Steinberg, and Frei 2023).

‘Inspired’ logics are most apparent in more programmatic genres such as TED talks. In these narratives, learning in traditional, standardized school contexts is problematized as demotivating, estranging, and not in keeping with our digital times, diagnoses which resonate well with the literature on the changing boundaries of learning (cf. Leander, Phillips, and Taylor 2010; Greenhow and Lewin 2016). TED talks on the future of learning technologies provide powerful examples of this narrative.^2^ Many of these talks refer to Ken Robinson’s powerful plea for overcoming remnants of the industrial age such as standardized teaching and testing in order to foster the free development of talent and creativity (Robinson’s talk is one of the most viewed TED talks ever).^3^ These visions are linked to widespread expectations of what students ‘really need to learn’ to be prepared for working and participating in digitized societies, skills ‘that cannot be adequately addressed by narrow and product-oriented views of education and schooling’ (Kumpulainen, Mikkola, and Jaatinen 2014, 53). They thus express a powerful rationale about the changing role of learning and (school) education in the digital era. They are also anchored in a changing political economy context (Boltanski and Chiapello 2018) which translates into ‘connectivist’ visions of learning.

The point is not whether these accounts are particularly coherent or convincing. The relevant point concerns the moral order they employ and promote: The purpose of learning and school education should be to further creativity, to develop problem-solving skills, grit, and entrepreneurship. Learning needs to be more social (furthering collaborative problem solving) and more personal at the same time: Learners should be acknowledged in their full individuality, their innate needs, interests, desires, and strengths shall guide the process of learning. Learning is therefore seen as a process that is best left to students themselves (Houlden and Veletsianos 2021). Learning environments should provide learning opportunities that allow students to create and follow their own individual trajectories, crossing boundaries between different learning contexts along the way as freely as possible.

This classificatory logic hence has far-reaching implications for how the boundaries of learning are conceived of and drawn, favouring no sharp rupture between informal and formal learning, between learning online and offline, or between learning across biographic contexts and stages. It also entails a clear hierarchy of relevant criteria for sorting and categorizing students: learners should be classified according to interests and talents, and always with the aim of improving the quality of ‘connectivity’ needed for team-oriented collaboration and creativity.

The more we move away from highly programmatic genres such as TED talks, the more the dominant classificatory logic shifts towards an industrial school world. Thus, blog posts on the possible uses of AI for everyday education (which are far more hands-on than the typical TED talk) typically emphasize the potential to increase efficiency, to automate the grading of papers, or to monitor students’ progress on their pathways through highly standardized learning contents and contexts.^4^ This industrial logic suggests that good and fair education is defined and realized through standardization, canonization, and efficiency.

Taking this classification logic seriously, we end up in stark opposition to the ideals of an inspired school world regarding how to sort and group students as well as concerning how the boundaries of learning are drawn. For example, following an industrial logic, we have strong arguments at hand for sticking to traditional curricula and standardized testing. There are also good reasons for retaining the division of learning content into traditional school subjects and of students into established categories such as age groups. In short, exactly the characteristics of industrial school worlds which are routinely denounced in TED talk after TED talk on the future of digital education.

A classification lens helps understand the persistence of these seemingly incoherent patterns in EdTech. Both industrial and inspired classificatory logics are deeply anchored in current education regimes and in narratives and dynamics of their digital transformation. Tension-ridden narratives should thus not be read as incoherent and insincere (which they of course might *also* be), but as compromises and complex configurations that – perhaps counter-intuitively – lend stability and durability to EdTech assemblages not despite but precisely because of their heterogeneity.

Both industrial and inspired logics of classification leave traces in understandings that actors develop about what an AI-enhanced future of learning might look like. They form part of the foundation on which actors become involved with new technologies in the first place and on which they establish a shared understanding about what digital learning should be all about.

### Tests – or: one-sided algorithms employing well established ‘statistical chains’

The second inspiration to be drawn from Desrosières’ sociology-of-numbers perspective on classification revolves around the heuristic of ‘tests’. This heuristic moves the focus from moral and epistemic orders to concrete algorithmic devices and how they perform classification tasks. With the heuristic of tests, our focus shifts from ‘conventional’ ideals of learning that actors rely on when drawing boundaries and making distinctions to how these ideals materialize in algorithms, data, and models inscribed into educational technologies. The heuristic of ‘tests’ centres our attention on the concrete design of digital tools, but also on the kind of (more or less bounded) learning experience a digital tool is designed for, or on how a concrete learning tool ‘harnesses’ information from different learning environments (crossing boundaries between learning contexts or not) when capturing and evaluating individual learning pathways.

The notion of tests plays a constitutive role for French pragmatic sociology which, in France, is actually often referred to as the ‘sociologie des épreuves’ – the ‘sociology of testing’ (Barthe et al. 2013). Latour employs the heuristic of tests in his laboratory studies which investigate procedures designed to decide who leaves scientific disputes as winner or as loser. Likewise, we can conceive of statistical classifications as a specific form of testing (Desrosières 2015; Desrosières and Thévenot 1979): as rules-based procedures that qualify some entity (human or non-human) according to some criterion or category. The heuristic of test underlines that any act of classification needs to ‘chain’ heterogeneous elements together into coherent and justifiable decision processes.

For our speculative inquiry into AIED, existing studies suggest a clear pattern regarding the kind of test they are designed to perform. This kind of test mirrors one side only of the hiatus diagnosed in the previous section: in line with an industrial logic of classification, learners are classified according to psychometric attainment scales following the example of large-scale assessments and standardized testing (Chang 2019).

Dixon-Román, Nichols, and Nyame-Mensah (2020) demonstrate this pattern for the example of adaptive essay writing tutors. Although the feedback to students is meant to be formative, it stays strictly within the limits of an ‘industrial logic’ of efficiently and effectively testing curricular knowledge and skills via standardized tests.

Perrotta and Selwyn (2020) confirm this tendency in their discussion of two prominent AI-based tutoring systems. The pattern also holds for algorithms that test for students’ emotional and motivational states to explain and evaluate learners’ engagement with standardized test items (McStay 2020; McStay and Rosner 2021). Further, Gulson, Sellar, and Taylor Webb (2022) discuss the role of AI-based face recognition software which classifies students into one of four states that clearly mirror an industrial understanding of teacher-centred instruction: ‘listening’, ‘writing’, ‘answering’, and ‘sleeping’ (!). Finally, Jarke and Macgilchrist (2021) show the same pattern for yet another kind of software – dashboards for classroom management which constitute causal narratives that entail evaluations and explanations of students’ learning progress very much in line with the logics of industrial school worlds.

The classificatory logic underlying these tests is completely out of tune with the narratives of creativity, grit, flexibility, and team working which are singled out as decisive skills for the digital era in ‘inspired’ school-world narratives. The one-size-fits-all approach of standardized testing is obviously not compatible with the idea of taking learning styles, students’ positional identities and lifeworlds, their interests and talents into account for a ‘true’ personalization of learning trajectories. The kind of classification performed also draws sharp boundaries regarding the relevance of formal versus informal learning contexts, the latter being devalued.

For software developers, the dominant test design does, however, have an important advantage: most of the ingredients it ‘chains’ together already exist in ready-made form. Test items, equivalence principles, justifications for the choice of achievement scales as indicators, models for calculating scales and assessing the difficulty of test items … all of these elements have been developed and established over the past decades in the context of test industries and large-scale assessment studies. The innovation task for the EdTech industry is thus very much simplified.

The analytic heuristic of tests helps identify a stark incongruence between the alleged goals of classification (that follow an ‘inspired’ vision of school education) and its ‘industrial’ implementation. Other forms of testing are in principle conceivable. To our knowledge, no devices have however so far been developed that are more in line with the current literature on the shifting purposes of learning and thus better aligned with the changing boundaries of learning in the digital era. Construing such ‘tests’ would of course be a demanding task. More radical AI-based learning tools would require information that spans and crosses learning contexts and allows to characterize learning networks and situations (Bricker and Bell 2014). Novel ‘statistical chains’ would have to be established that start with collecting new kinds of information or using existing data for other ends.

Thinking about classification in terms of conventional logics and of testing devices furthers our understanding of how sorting algorithms in current AIED are designed, and why. To better understand the actual sorting effects of digital learning tools, we need to add a third dimension to our classification lens. This third dimension focuses on the *situations* in which digital learning tools are employed.

### Situations – or: the threat of invisibilized social sorting in digital learning

The choice of ‘situation’ as third conceptual anchor for reflection and analysis mirrors a fundamental orientation of French pragmatic sociology. The analytical focus is on how social actors – always conceived of as capable and knowledgeable (Boltanski and Thévenot 1999) – engage with objects and discourses in order to define and handle uncertain situations. Following a pragmatic understanding, the term situation here refers to configurations of actors and objects that require some sort of ‘handling’. Confronted with uncertainties and tensions, actors must define a situation as presenting a specific kind of problem which they then can deal with drawing on available knowledge and resources. Understood in this sense, students face a specific kind of ‘situational problem’ when learning, and teachers face another kind when creating learning opportunities.

A strong general orientation to ‘situations’ is evident in how Desrosières discusses processes of statistical classification, from the emergence of statistical categories in particular historical and political situations (Desrosières 2016), to how social actors interpret and negotiate the meaning of these categories in everyday situations (Desrosières and Thévenot 1979), to how numbers develop performative effects in concrete situations (Desrosières 2015).

The heuristic of situations sensitizes us for the complexity of the interplay between classification devices and social sorting as they materialize in concrete learning settings. A focus on situations further allows to take students’ positional identities (Roth and Erstad 2016) and teachers’ pedagogical understandings into account for unravelling how digital tools and the learning environments built around them might affect learning trajectories in more or less equitable ways (Chavez 2021). On this basis, the pragmatic notion of ‘situations’ allows to understand how the combination of different strategic agencies that actors (students, teachers, parents, …) with their respective self-imaginations and problem understandings develop in a given situation can lead to unforeseen consequences.

Returning to our speculative inquiry, such an orientation to situations leads us to formulate two conjectures that emerge from existing scholarship and our own empirical engagement with digital learning tools thus far:

(1) Both ‘inspired’ and ‘industrial’ classification dispositifs can be anticipated to reinforce existing patterns in learning trajectories, but in different ways. For the ‘industrial’ case, the dynamics we must expect are well understood (Chang 2019; Mann and Matzner 2019; Leavy 2018). An industrial logic suggests that students are sorted according to their performance on standardized test items defined according to traditional curricular canons. Since these canons mirror dominant epistemic orders, they are inevitably socially biased. Speculating about such dynamics in (future) digital learning environments, already biased algorithmic sorting may develop into vicious circles with important feedback effects. Students being classified and addressed as weak or strong by some kind of digital learning tool are likely to develop corresponding self-understandings. Biased data thus threaten to turn into self-fulfilling predictions of educational success or failure.

For the ‘inspired’ scenario, other mechanisms of self-reinforcement can be expected. Even if inspired logics of classification have so far not been translated into designs for algorithmic testing (see above), these sorting dynamics matter for our speculative inquiry into digital learning. After all, regardless of the kind of digital tool employed, learning *environments* may very well be structured according to the logics of an inspired school world. In these sorts of learning environments, students’ positional identities – and their resulting ways of perceiving, evaluating, and handling educational situations – become decisive. While the industrial logic of classification is presumably already causing troubling sorting dynamics *within algorithmic black-boxes*, inspired logics may develop problematic effects in the *black-boxes’ surroundings*.

Patterns of self-sorting may emerge in digital learning environments that follow an inspired school world logic because learners engage with these tools on the basis of their individual biographies and positional identities. ‘Creative’ digital learning environments can surely make learning trajectories more flexible, but there is the very real possibility of reinforcing sorting dynamics along social categories such as gender, class, or ‘race’. In ethnographic research in our own projects, we for example encountered strikingly uniform gender patterns in in-school learning settings that emphasized self-regulated and authenticity-oriented ‘creative’ school world logics. Among others, there was an almost ‘perfect’ self-sorting regarding ICT-related courses, with a clear dominance of boys. There is quantitative evidence that this self-sorting may be a more general pattern. Thus, in ICILS 2018 data for Germany, girls tend to outperform boys on ICT-related psychometric achievement scales. Seen through a classification lens, they are categorized as doing well in ‘industrial’ terms. At the same time, they appear distinctly less ‘ICT-savvy’ if we switch to variables that are more akin to self-regulated dynamics of inspired school worlds: girls are far less likely to visit non-obligatory ICT-related courses, less prone to envision an ICT-related future for themselves, and tend to assess their ICT-related self-efficacy more negatively.

(2) The crucial question then is how these potential dynamics of (self-)sorting can be detected and addressed within and across concrete learning environments. At this point, our classification lens comes full circle: whether and how actors problematize situational sorting dynamics and how they react to them will depend on the classificatory logic they draw on (resp. on the understanding of educational quality and justice they mobilize in a given learning situation). At this point, our first conjecture of likely sorting dynamics needs to be linked to a second conjecture: in concrete situations of learning and teaching, there is a high risk that dynamics of social sorting are systematically invisibilized, both technologically and pedagogically.

Whitman (2020) provides an impressive example for the kind of invisibilization dynamics that can be expected for ‘industrial’ classification scenarios. She discusses how software engineers are reluctant to include social categories such as ‘race’ or gender into their algorithms to control for possible social selectivity because students themselves cannot change these variables. Including such ‘attributes’ into predictive models that follow standardized test logics would therefore ‘not seem fair’. Within the limits of an industrial understanding of merit and learning, this makes absolute sense: learning is imagined as a process that can and must be managed and monitored by standardized instruments that, by definition, are imagined to be neutral and blind towards social categories.

For inspired school worlds, we must also suspect dynamics of de-thematization, even if a different kind. Regarding our own empirical findings on gender-related self-sorting (from qualitative interviews conducted during the pilot study to our SNSF-project, see above), teachers repeatedly reacted surprised when we confronted them with the apparent fact of pronounced gender patterns in their mobile/flexible/self-regulated learning environments. They just had not noticed. More importantly, they were uncertain whether these sorting patterns should be seen as problematic or not. If learning is truly about what learners themselves strive to do and to become, who are we to problematize (or even worry about) their choices? These (potentially troubling) patterns of de-thematization can seem fully understandable and reasonable within an ‘inspired’ logic of classification.

The concrete sorting effects that digital learning tools develop thus hinge on a complex interplay of technological designs, learning environments, and students’ biographies and identities. The heuristic of situation underlines this interplay, emphasizing the constitutive role of learners for the formation of learning experiences and pathways. Conventional logics of classification inform how teachers and other actors perceive and evaluate the resulting dynamics, and how they reason about possible adjustments to digital learning environments.

## Conclusion

This article started from the question how classification theory could advance our understanding of social sorting dynamics in digital learning contexts. Our key argument is that a classification lens allows to bridge uncertain situations of coordination with overarching societal moral and epistemic orders. Thus, different understandings of what makes ‘good and fair school education’ become effective in how actors ‘on the ground’ define and design both digital tools and learning environments, how they draw boundaries around them, and what boundary-crossing relations to other learning contexts (including learners’ lifeworlds) they find valuable and adequate.

Inspired by Desrosières' sociology of numbers, we propose a framework for grasping such configurations that entails three guiding heuristics which operate on different analytical levels: *conventions*, *tests*, and *situations* of classification. The key task for social research is to look at the interlocking of these three dimensions. To demonstrate the productivity of this heuristic framework, we presented a speculative inquiry into the classificatory dynamics of AIED; this brief exemplary study sheds light on a crucial hiatus between two logics of classification that affect current learning technologies, one ‘inspired’, the other ‘industrial’. Both these logics develop important effects – be it by structuring algorithmic tests (mostly ‘industrial’), be it by informing how social actors envision, enact, and evaluate situations of learning in the digital era (often ‘inspired’). The interplay of classificatory logics and algorithmic tests in concrete situations may lead to unforeseen sorting dynamics that emerge on the basis of students’ pre-existing learning trajectories and positional identities. These dynamics are particularly troubling if they are invisibilized due to the dominance of classificatory logics that hinder their problematization.

The applicability of such a classification lens is easy to see in the case of AI algorithms which our discussion focused on. However, a classification lens can be fruitfully employed for debating or analysing the social foundations and consequences of any digital learning tool. Even if the technology at stake does not employ sorting algorithms, its overall design and its implementation are inevitably informed by classificatory logics. For example, decisions regarding the aesthetics and functionality of user interfaces are never purely technical issues but must also be compatible with the categories and criteria that teachers and learners use to define and handle learning situations. Be it the choice of learning content and test formats, the way learners are represented and addressed in digital environments, or the communication and cooperation functionalities built into a digital learning tool, educational technologies are through and through informed by classificatory logic allowing to distinguish ‘relevant’ from ‘irrelevant’, ‘adequate’ from ‘inadequate’, or ‘fair’ from ‘unfair’. What is more, they are always employed in situations that are designed, monitored, and evaluated according to some educational classificatory logic. To put the dimension of classification centre stage – to investigate where classificatory logics leave their marks, to ask about their possible consequences, and to inquire into how different classificatory dynamics become interlocked in the shaping of trajectories and identities within and across learning contexts – opens new perspectives on dynamics of social sorting in digital learning.

The task of ‘rethinking the boundaries of learning in a digital age’ thus emerges as a double challenge: First, we need to understand how the design and usage of digital learning tools depend on how social actors (students, teachers, and engineers) imagine and constitute boundaries and orders of learning. Second, we need to unravel how these very bounded learning contexts can result in unwanted and unforeseen sorting dynamics. A classification lens can help understand why such unwanted patterns can persist – not *although* but *because* they are full of tensions and anchored in countervailing ideas of what makes good and fair school education.

## Notes

1 For more information, see the project description in the Swiss National Science Foundation Project Database: https://data.snf.ch/grants/grant/204737 [23 April 2023].

2 Scott Boland provides a typical example for the genre: https://www.youtube.com/watch?v=cYIvfS-knA [23 April 2023].

3 https://www.ted.com/talks/sir_ken_robinson_do_schools_kill_creativity [23 April 2023].

4 A more or less random example: https://www.uopeople.edu/blog/ai-in-education-where-is-it-now-and-what-is-the-future/ [23 April 2023].
